# Supplementary material for: The influence of subglacial lake discharge on Thwaites Glacier ice-shelf melting and grounding-line retreat
Source: Nat Commun. 2025 Mar 6;16:2272. doi: 10.1038/s41467-025-57417-1 (PMC11885594; doi:10.1038/s41467-025-57417-1)
Supplement: Supplementary file 1 — Supplementary Information [file 41467_2025_57417_MOESM1_ESM.pdf]

Supplementary information

1. Active subglacial lake discharge timeseries

In addition to the 4 already documented active subglacial lakes, we identified a further 3 lakes showing activity during the 2013 lake discharge episode, lake Thw44, Thw270, and Thw408 (Figure 1, Figure S1). Lake Thw270 drained about 1 km^3, lakes Thw44 and Thw408 drained 0.3 km^3 and 0.1 km^3 respectively.

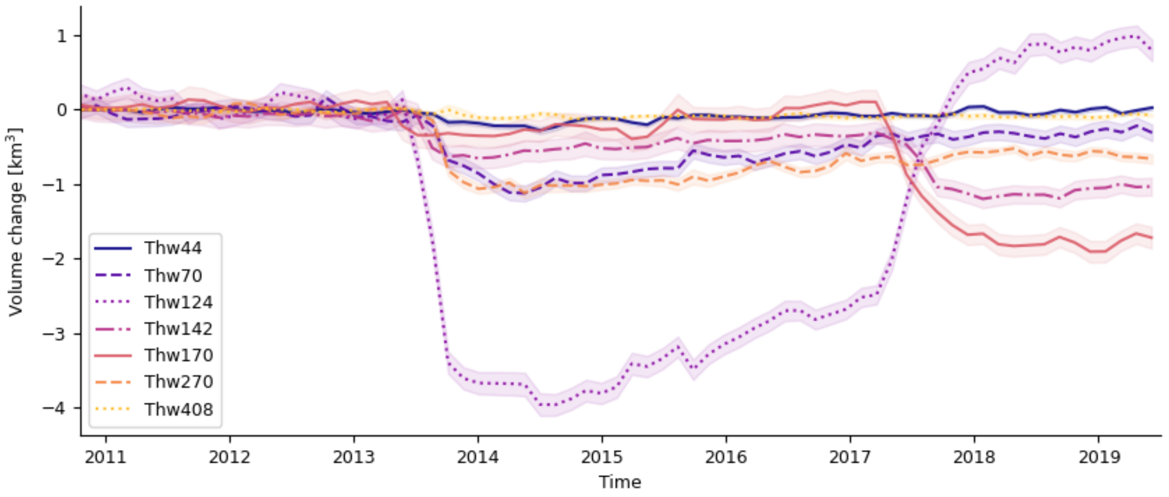

Figure S1: Volume change timeseries over the 7 subglacial lakes identified as being active in 2013. The number following "Thw" indicates distance of the lake from the grounding line.

2. Basal melt time-series

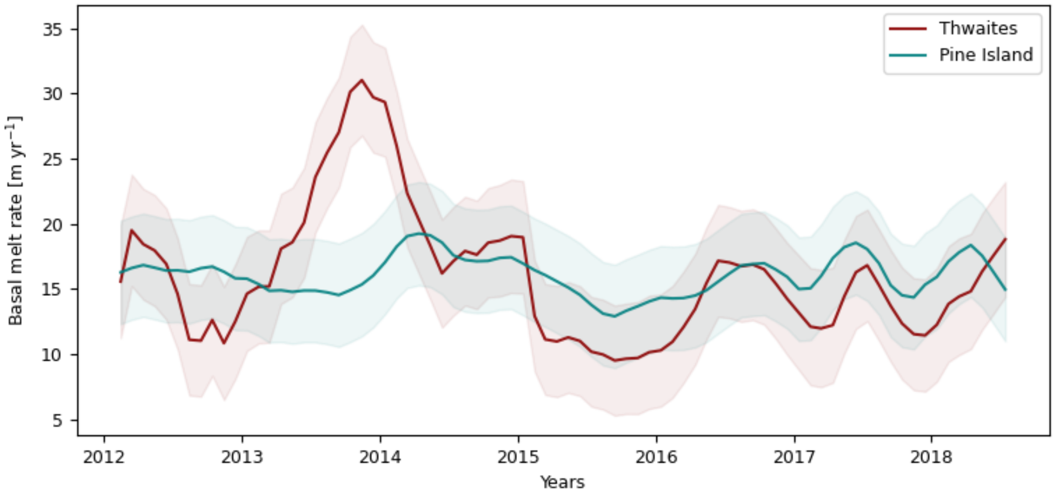

Figure S2: Basal melt time-series for Thwaites and Pine Island ice shelves (see figure 1 for location and extent of sectors). "Thwaites" corresponds to the sector including both TEIS and TWITgl.

3. Ice shelf basal melt rate anomaly components

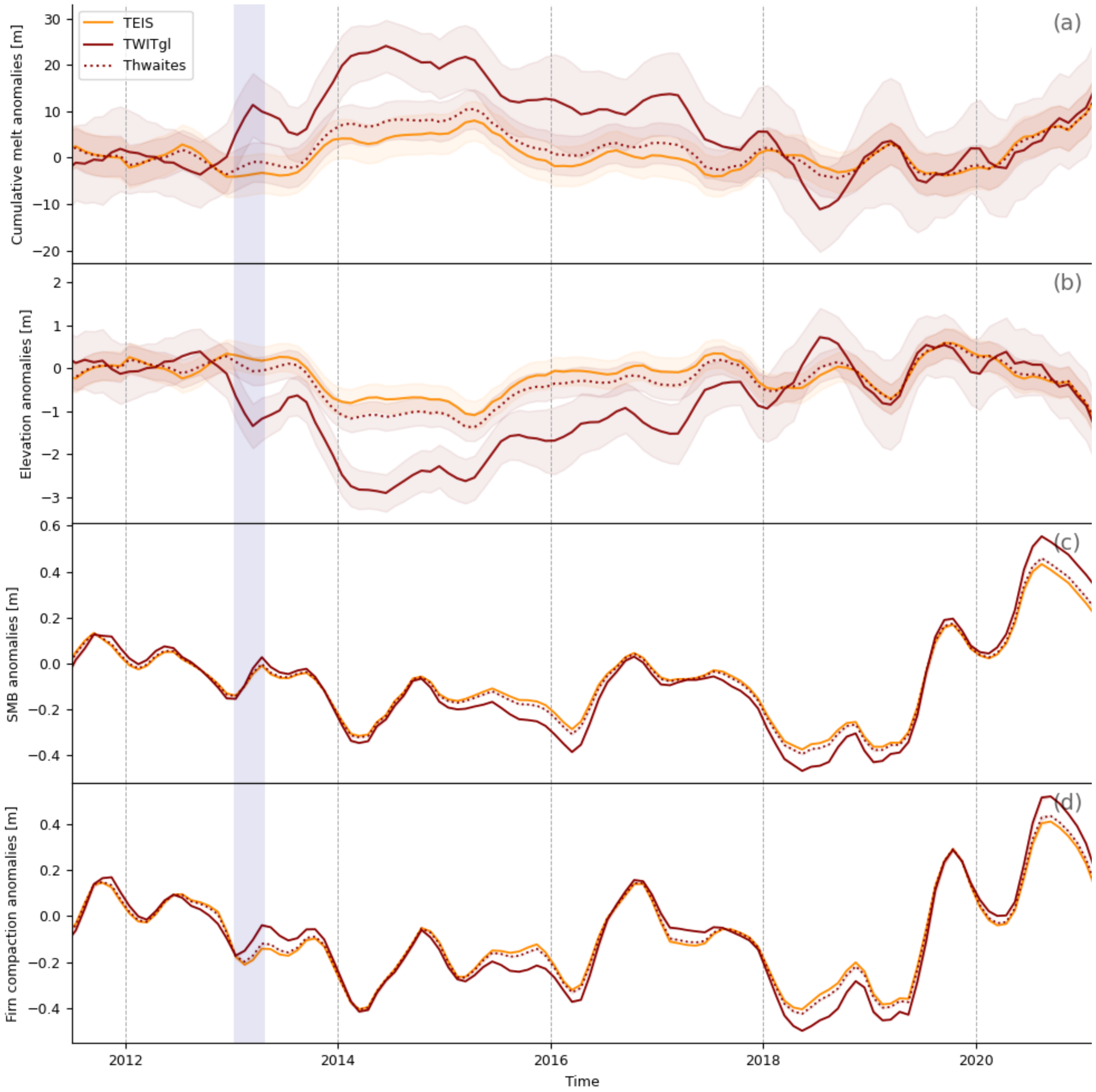

Figure S3: Basal melt and components used for its derivation. Cumulative melt anomaly, "Thwaites" corresponds to the sector including both TEIS and TWITgl (see figure 1), (a), surface elevation anomaly (b), Surface Mass Balance anomaly (c), Firn compaction anomaly (d). The vertical lilac shading across all 5 plots marks the onset of lake drainage taking place sometimes between January and April 2013[1,2].

Temporal variability in calculated basal melt rate derives from variability in elevation, Surface Mass Balance, and firn compaction. See section b. for discussion on ice velocity. Elevation change drives in large parts the basal melt variability, variability of surface components is an order of magnitude lower.

4. Draft depth

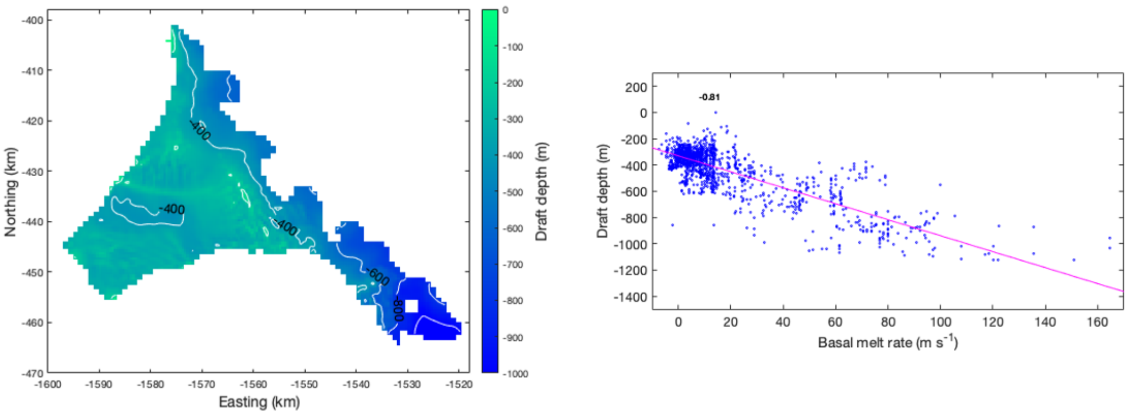

Figure S4: Depth of the ice draft (D)[3] over TEIS and TWITgl (left), and correlation (Pearson correlation of 0.81, Pvalue of 0) between D and basal melt rates $\dot{m}$. Linear rate shown in pink corresponds to $\dot{m} = -54.1 - 0.1642D$.

5. Variability within TEIS

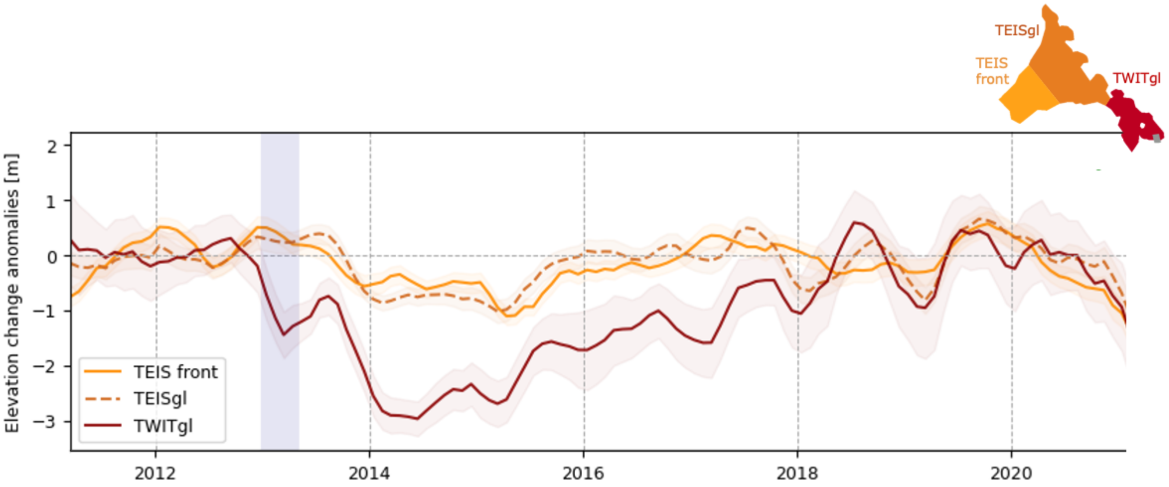

Figure S5: Time series of elevation change anomalies. Same as figure 2b but with TEIS split into two sectors with distinct distance from the grounding and distinct ice draft, showing little differences in elevation anomaly.

6. Elevation change and hydrostatic equilibrium assumption near the grounding line

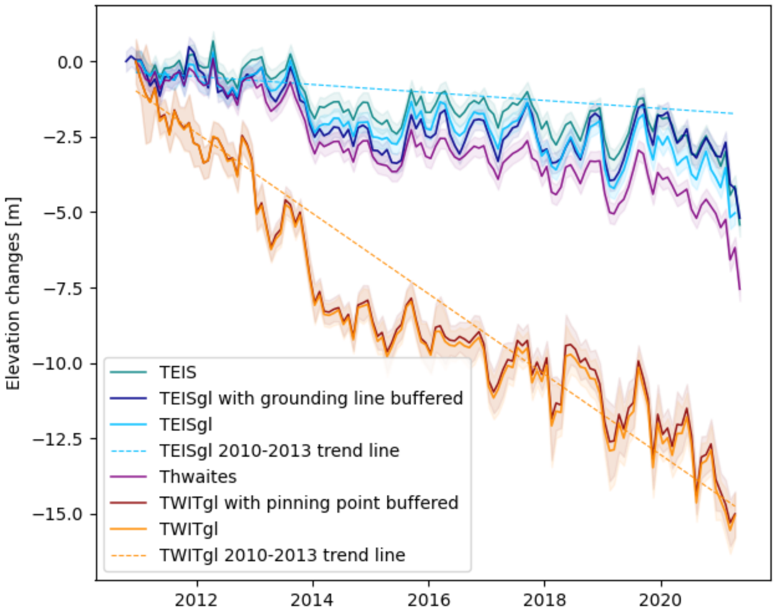

Figure S6: Elevation change for the various Thwaites sectors of floating ice discussed in the study. Impact of excluding ice shelf near the grounding line and the pinning point on change in surface elevation is shwon. We consider a 5 km exclusion zone for the grounding line, and 2km for the pinning point i.e. 3 times the ice thickness. The impact is relatively minor at the scale of the chosen sectors. Dashed line is the linear regression through the 2010-2013 period, prior to the 2013 thinning.

7. Hydrographic moorings

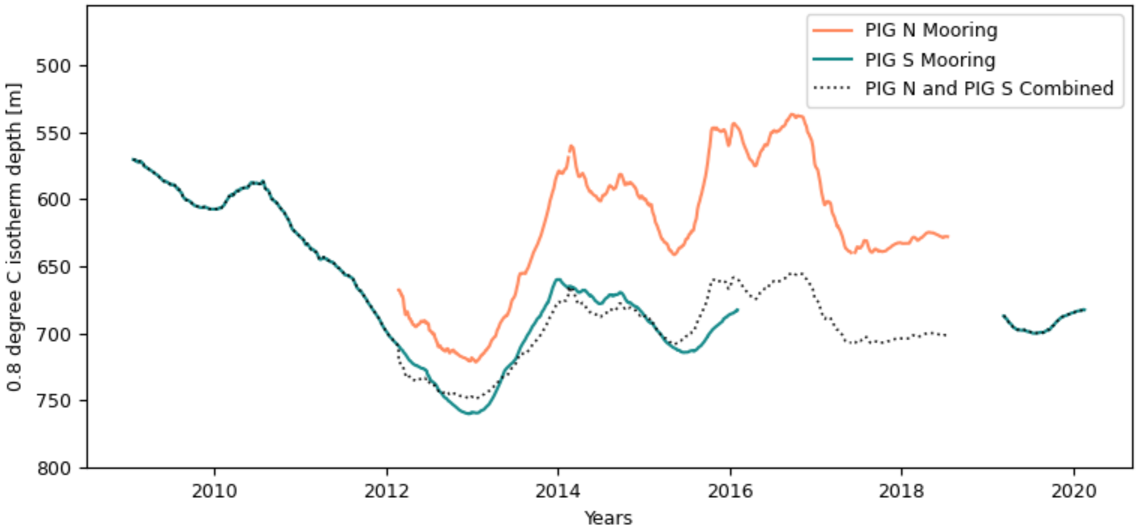

Figure S7: Timeseries for the individual hydrographic moorings used to derive the thermocline depth shown in Figure 2e (black dashed line).

8. MODIS images of the Thwaites western ice tongue polynya

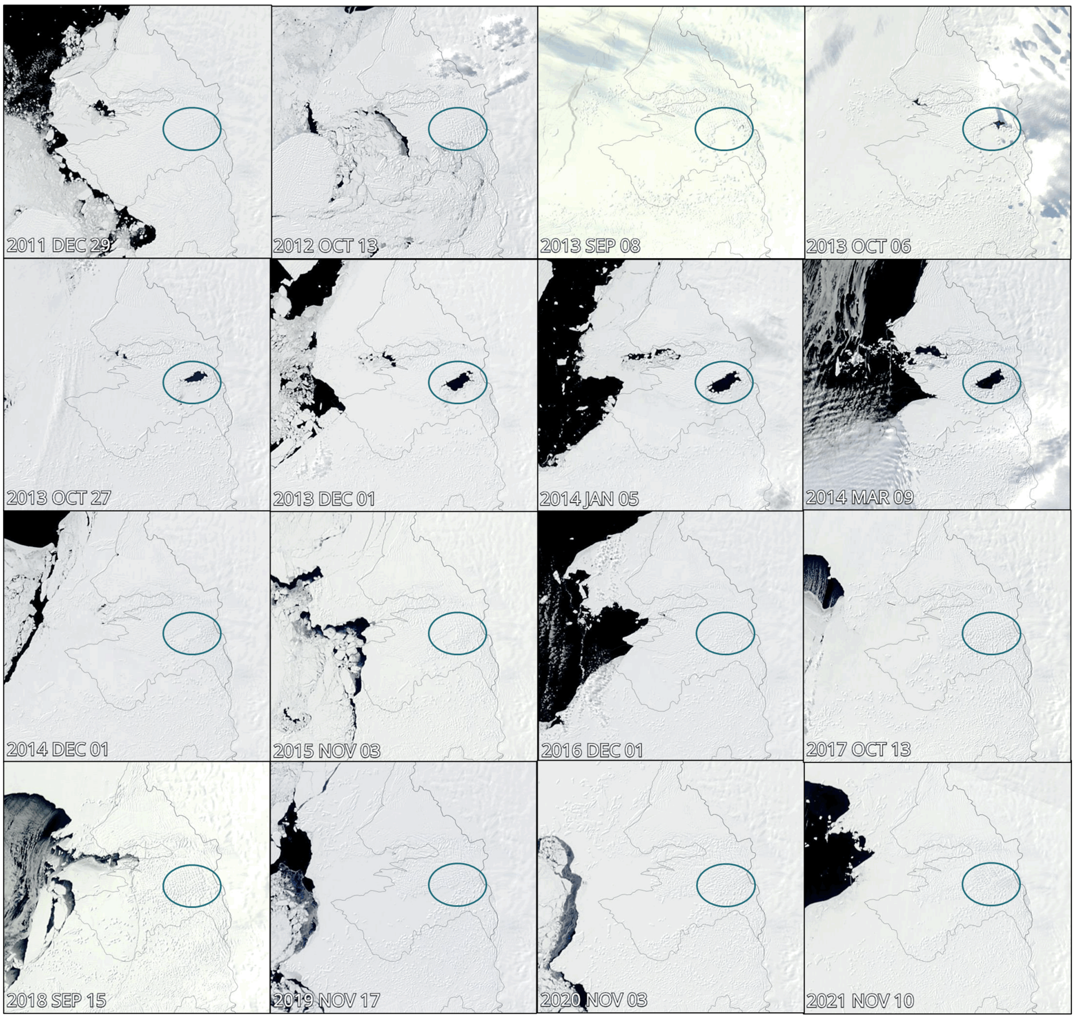

Figure S8: Time-series of MODIS image in the Thwaites sector between 2011 and 2021. It illustrates the formation of a localised Polynya (indicated by blue circle) in 2013 near the TWITgl sector starting in September 2013, this was an exceptional event during the 2011-2021 period. These are NASA Worldview Snapshots hence the outlines differ from those shown in e.g. Figure 1 of the main manuscript.

9. Change in elevation and velocity at the TWIT grounding line embayment

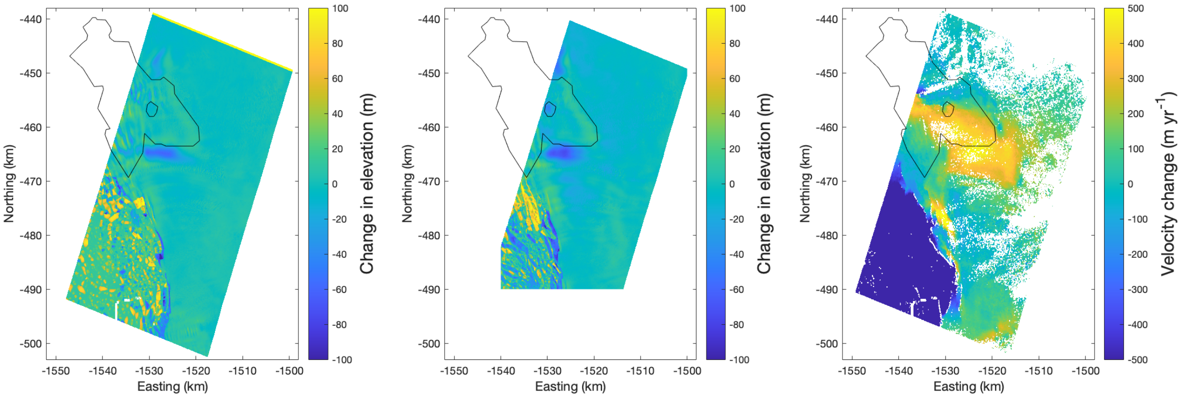

Figure S9: Grounded ice – floating ice transition in the TWIT sector. Rapid change in surface elevation and velocity near the area of the 2013 ungrounding (centre of the image) Left: between December 2013 and July 2017 (Fig. 1 of [4]), middle: between June 2011 and December 2018[5], used to determine the extent of the area over which elevation timeseries in

Figure 2c is calculated. Right: change in ice velocity between January 2012 and January 2020 (Fig. A2 of [4]). The black polygon in the 3 images depicts the boundary of the TWITgl sector (Figure 2a).

10. Lag between Thwaites basal melt rate and subglacial flux

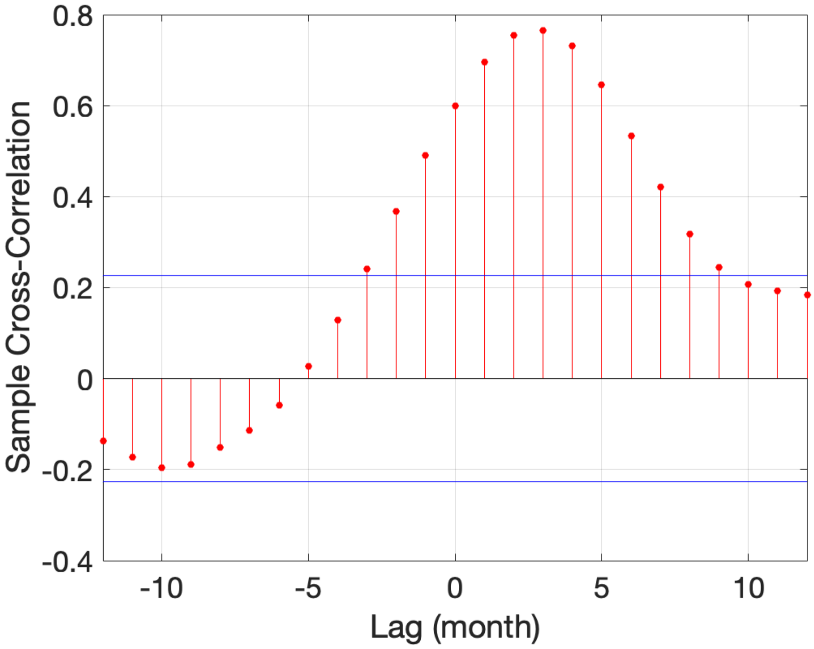

Figure S10: Variation in Pearson correlation between Thwaites' basal melt rate and subglacial flux. Maximum correlation is found for a lag of 3 months (Figure 3).

11. Modeling the impact of rapid subglacial discharge on ocean and ice shelf basal melt rate

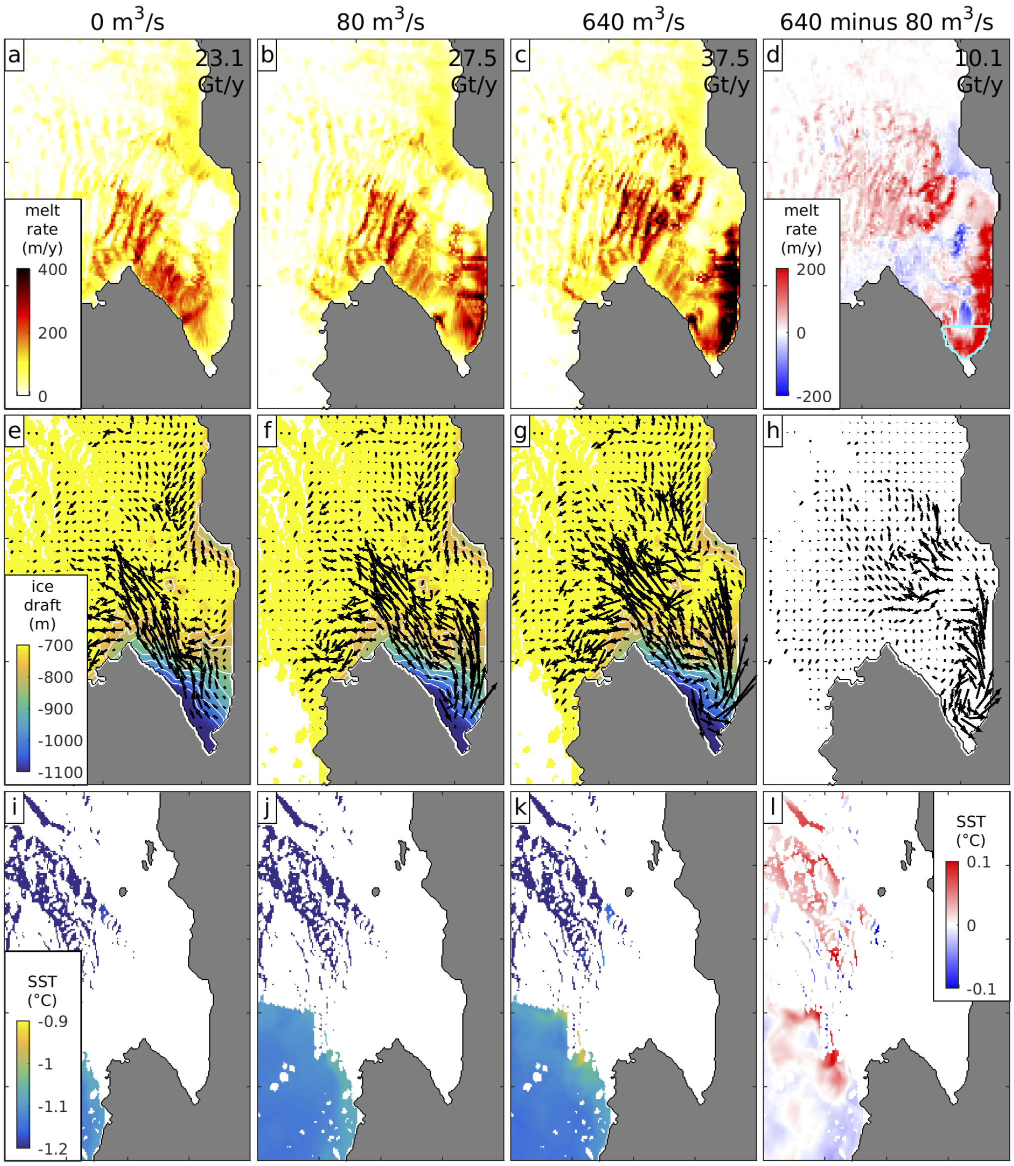

Figure S11: Modelled melt rates, ocean circulation, and Sea Surface Temperature (SST), near the TWITgl sector (Figure 2a). Location of imposed subglacial lake outflow is indicated by the cyan semi-circle in panel d). The rows show melting, ice draft and sub-ice currents, and SSTs. The columns show results for no subglacial discharge (a, e, i), a subglacial discharge of 80 m³/s corresponding to the expected steady-state subglacial discharge (b, f, j), a subglacial discharge of 640 m³/s corresponding to the combined peak discharge during lake drainage (c, g, k), and the difference between the peak discharge and the steady-state (d, h, l). Note that the spatial pattern of predicted melt, in particular its location along the grounding line, is uncertain as it is strongly influenced by the shape of the cavity, which under Thwaites ice shelf, is poorly known

12. CryoSat-2 data coverage

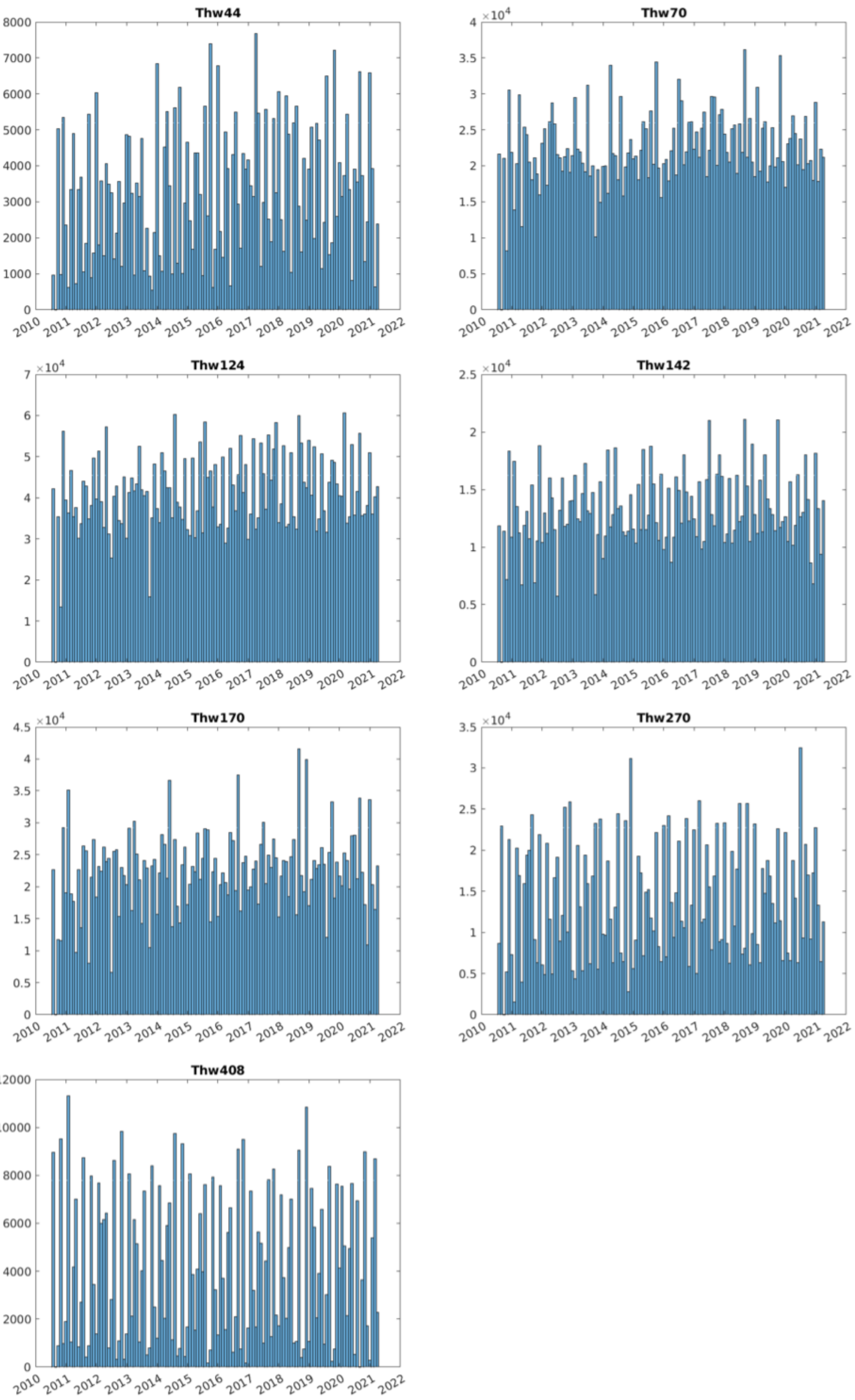

Figure S12: Number of CryoSat-2 elevation measurements falling into each of the seven subglacial lake perimeters every month.

13. Impact of using a constant ice velocity

Change in ice velocity and ice thickness impact the calculation of basal melt via the calculation of ice advection and ice divergence. In this work we apply a constant velocity field to the 2011-2020 period for both the advective and divergence term of the basal melt equation, the velocity field is from ITS_LIVE[6,7]. The choice of a single velocity composite is driven by the fact that ice velocity products are more robust from 2014 onward, that ice velocity has remained relatively constant during the time-period at TEIS, that the changes in elevation and velocity observed near TWIT's grounding line are localised and not sufficient to significantly impact the calculation, that observed elevation change would require unrealistic velocity change.

Velocity variation near the TWIT grounding line

During the TWIT grounding line retreat of 2013, ice velocity increased and thinning took place, by 2020 ice velocity increase by up to 15%[4] (Figure S9). Taking into account the change in velocity and thickness between 2013 and 2020 when calculating basal melting changes the melt rate estimate only by 5.3% over the TWITgl sector. Note that the observed speed-up and thinning took place progressively between 2013 and 2020, the velocity change in 2013 and 2014 was significantly lower than 15%[4].

Velocity quality & known velocity variation

Before 2013 and the launch of Landsat-8, the accuracy and temporal resolution of ice velocity product in this sector is relatively poor. The availability of Landsat-8 improved accuracy but only during the summer months and when cloud cover is favourable. From 2015 and the launch of Sentinel-1 ice velocity could be obtained all year long, even during the polar winter. Analysis of the existing products, at annual resolution, reveal relative stability of the TEIS velocities since 2011[8], with even during rapid change in the velocity structure of the remaining of the TWIT area[9] (Figure S13).

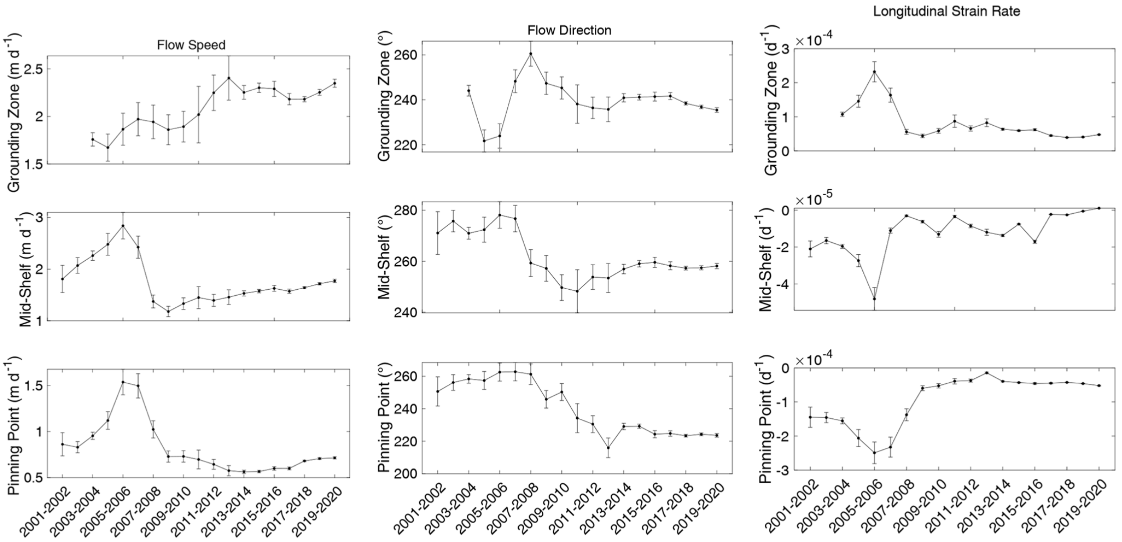

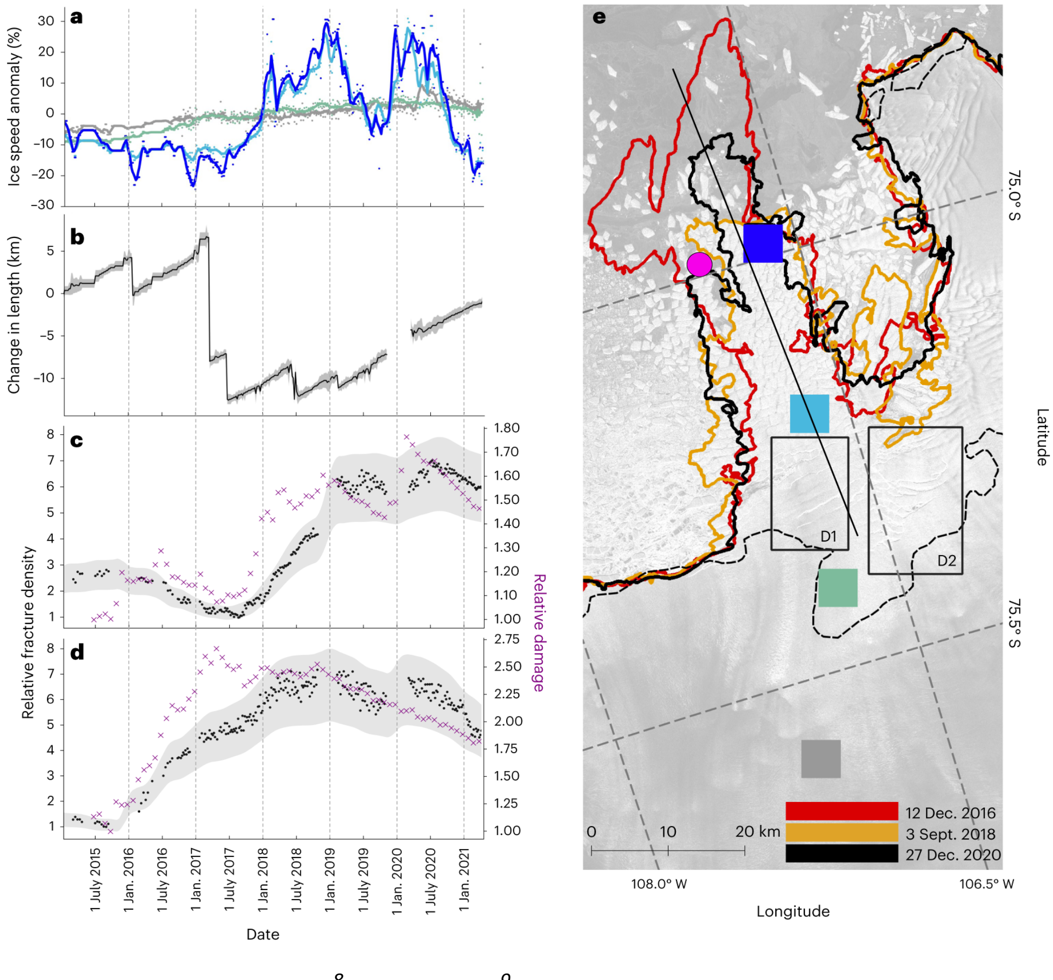

Figure S13: Ice velocity change over TEIS[8] (left), and TWIT[9] (right). Since 2011, annual velocity has been relatively constant over TEIS. Despite rapid change downstream of TWIT, the TWIT sector close to the grounding line sees relatively little (i.e. ~10%) velocity variation.

Change in velocity and thickness required to explain the observed 2013 thinning

Further to the impact of observed changes on determining the melt rate, we also calculate the change in velocity needed to explain the observed change in ice thickness of TWITgl, TEISgl, and TEIS via advection and divergence.

Advection

Ice flow brings thicker ice downstream, a slowdown in ice advection therefore could lead to the observed ice shelf thinning. Given the slope of the thickness profile of 3.5%, 1%, and 1% and mean velocity of 1,306 m a^{-1}, 575 m a^{-1}, and 697 m a^{-1} respectively for TWITgl, TEISgl, and TEIS, advection alone adds 46 m, 6 m, and 7 m of ice each year to TWITgl, TEISgl, and TEIS. For advection to explain the observed annual thickness change, ice velocity would need to decrease by 35% TWITgl, and would be an impossibility for TEIS and TEISgl as the required deceleration is larger than the current ice velocity (Figure S14). These values are unrealistic given our knowledge and understanding of ice flow at Thwaites glacier.

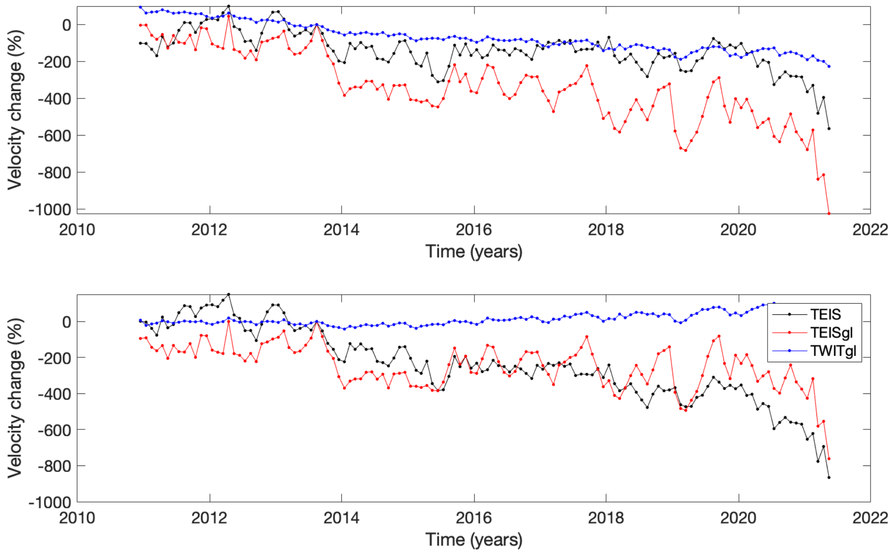

Figure S14: Velocity change needed to explain the observed elevation change via change in ice advection. Total elevation change (top), elevation change anomaly (bottom). Vertical reference is set to the month of July 2013 when the drop in elevation occurs.

Divergence

Ice flow divergence over ice shelves acts to reduce ice thickness. An acceleration of the glacier, leading to an increase in flow divergence, could therefore cause ice shelf thinning. Over Thwaites currently, ice divergence accounts for 11.2 m yr^{-1}, 4.1 m yr^{-1} and 1.6 m yr^{-1} of vertical thinning for the TWITgl, TEISgl, and TEIS sectors respectively. For divergence to explain the observed thickness change, ice velocity would need to increase by 174%, 520%, and 670% respectively over the TWITgl, TEISgl, and TEIS sectors (Figure S15). These values are unrealistic given our knowledge and understanding of ice flow at Thwaites glacier.

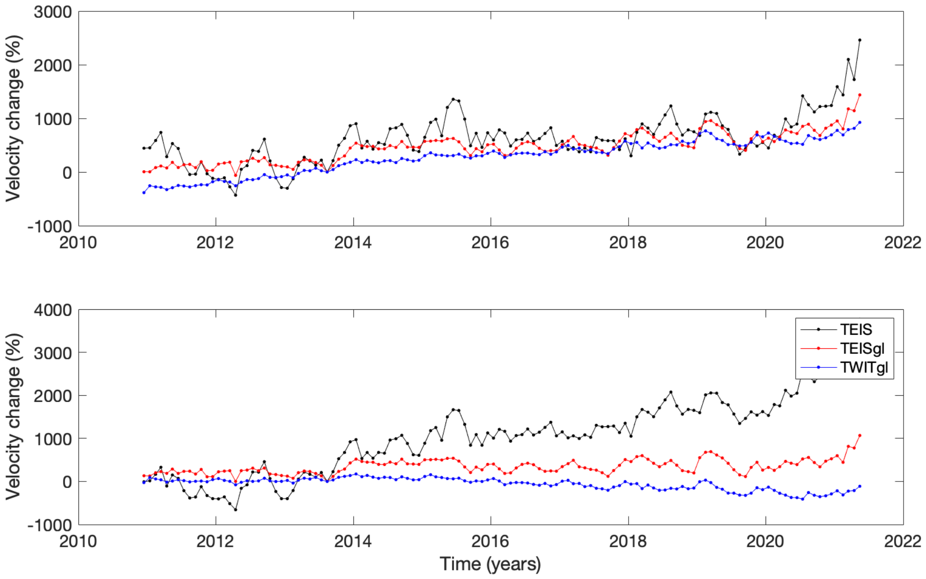

Figure S15: Velocity change needed to explain the observed elevation change via change in ice divergence. Total elevation change (top), elevation change anomaly (bottom). Vertical reference is set to the month of July 2013 when the drop in elevation occurs.

14. Impact of change in volume scattering

Volume scattering can lead to apparent change in surface elevation from radar altimetry by impacting the depth of the scattering phase centre[10]. The 2013 elevation change observed over TEIS and TWITgl is unlikely to be related to change in volume scattering because i) TFMRA retracking is known to be robust against volume scattering change even during exceptional changes in surface conditions[11], ii) rapid changes in volume scattering are associated with presence of liquid water leading to an upward shift in the scattering horizon and thus an apparent increase in surface elevation, the opposite of what is observed in 2013, iii) such change in scattering depth are usually correlated over large areas, however the change in elevation observed here is only taking place over Thwaites floating ice, and not in the rest of the Amundsen sector.

1. Smith, B. E., Gourmelen, N., Huth, A. & Joughin, I. Connected subglacial lake drainage beneath Thwaites Glacier, West Antarctica. *The Cryosphere* **11**, 451–467 (2017).
2. Malczyk, G., Gourmelen, N., Goldberg, D., Wuite, J. & Nagler, T. Repeat Subglacial Lake Drainage and Filling Beneath Thwaites Glacier. *Geophys. Res. Lett.* **47**, (2020).
3. Morlighem, M. *et al.* Deep glacial troughs and stabilizing ridges unveiled beneath the margins of the Antarctic ice sheet. *Nat. Geosci.* **13**, 132–137 (2020).
4. Bevan, S. L., Luckman, A. J., Benn, D. I., Adusumilli, S. & Crawford, A. Brief communication: Thwaites Glacier cavity evolution. *The Cryosphere* **15**, 3317–3328 (2021).

5. Holland, P. R., Bevan, S. L. & Luckman, A. J. Strong Ocean Melting Feedback During the Recent Retreat of Thwaites Glacier. *Geophys. Res. Lett.* **50**, e2023GL103088 (2023).
6. Adusumilli, S., Fricker, H. A., Medley, B., Padman, L. & Siegfried, M. R. Interannual variations in meltwater input to the Southern Ocean from Antarctic ice shelves. *Nat. Geosci.* **13**, 616–620 (2020).
7. Gardner, A. S. *et al.* Increased West Antarctic and unchanged East Antarctic ice discharge over the last 7 years. *The Cryosphere* **12**, 521–547 (2018).
8. Alley, K. E. *et al.* Two decades of dynamic change and progressive destabilization on the Thwaites Eastern Ice Shelf. *The Cryosphere* **15**, 5187–5203 (2021).
9. Surawy-Stepney, T., Hogg, A. E., Cornford, S. L. & Davison, B. J. Episodic dynamic change linked to damage on the Thwaites Glacier Ice Tongue. *Nat. Geosci.* **16**, 37–43 (2023).
10. Nilsson, J. *et al.* Greenland 2012 melt event effects on CryoSat-2 radar altimetry. *Geophys. Res. Lett.* **42**, 3919–3926 (2015).
11. Nilsson, J., Gardner, A., Sandberg Sørensen, L. & Forsberg, R. Improved retrieval of land ice topography from CryoSat-2 data and its impact for volume-change estimation of the Greenland Ice Sheet. *The Cryosphere* **10**, 2953–2969 (2016).
