# Supplementary figures and images for: The influence of subglacial lake discharge on Thwaites Glacier ice-shelf melting and grounding-line retreat
Source: Nat Commun. 2025 Mar 6;16:2272. doi: 10.1038/s41467-025-57417-1 (PMC11885594; doi:10.1038/s41467-025-57417-1)

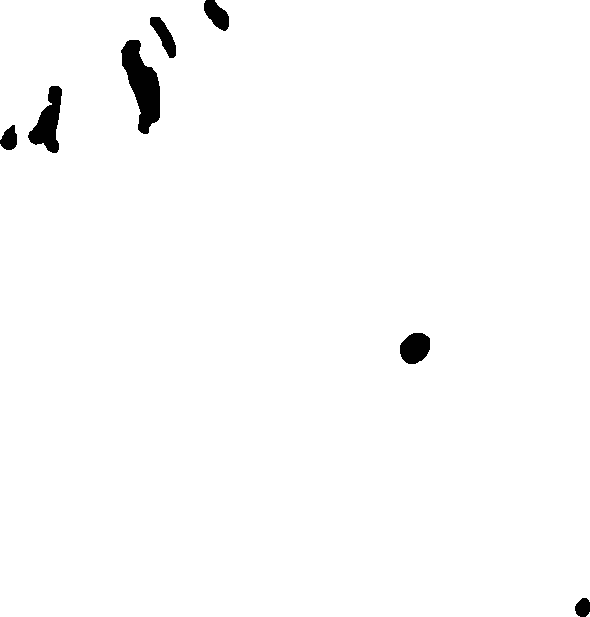

Supplement: Supplementary file 4 — Source Data 2 [file 41467_2025_57417_MOESM4_ESM.zip › SourceData/1a_subglacial_lake_mask.tif]
